# Phenotypic and genetic alterations of *Burkholderia pseudomallei* in patients during relapse and persistent infections

**DOI:** 10.3389/fmicb.2023.1103297

**Published:** 2023-02-06

**Authors:** Rathanin Seng, Rungnapa Phunpang, Natnaree Saiprom, Adul Dulsuk, Claire Chewapreecha, Janjira Thaipadungpanit, Elizabeth M. Batty, Wasun Chantratita, T. Eoin West, Narisara Chantratita

**Affiliations:** ^1^Department of Microbiology and Immunology, Faculty of Tropical Medicine, Mahidol University, Bangkok, Thailand; ^2^Mahidol-Oxford Tropical Medicine Research Unit, Faculty of Tropical Medicine, Mahidol University, Bangkok, Thailand; ^3^Parasites and Microbes, Wellcome Sanger Institute, Cambridge, United Kingdom; ^4^Department of Clinical Tropical Medicine, Faculty of Tropical Medicine, Mahidol University, Bangkok, Thailand; ^5^Nuffield Department of Medicine, Centre for Tropical Medicine and Global Health, University of Oxford, Oxford, United Kingdom; ^6^Center for Medical Genomics, Faculty of Medicine Ramathibodi Hospital, Mahidol University, Bangkok, Thailand; ^7^Division of Pulmonary, Critical Care, and Sleep Medicine, Department of Medicine, University of Washington, Seattle, WA, United States; ^8^Department of Global Health, University of Washington, Seattle, WA, United States

**Keywords:** *B. pseudomallei*, melioidosis, recurrent infection, relapse infection, persistent infection, whole-genome sequencing, within-host mutation, within-host alterations

## Abstract

The bacterium *Burkholderia pseudomallei* is the causative agent of melioidosis, a severe tropical disease associated with high mortality and relapse and persistent infections. Treatment of melioidosis requires prolonged antibiotic therapy; however, little is known about relapse and persistent infections, particularly the phenotypic and genetic alterations of *B. pseudomallei* in patients. In this study, we performed pulsed-field gel electrophoresis (PFGE) to compare the bacterial genotype between the initial isolate and the subsequent isolate from each of 23 suspected recurrent and persistent melioidosis patients in Northeast Thailand. We used whole-genome sequencing (WGS) to investigate multilocus sequence types and genetic alterations of within-host strain pairs. We also investigated the bacterial phenotypes associated with relapse and persistent infections, including multinucleated giant cell (MNGC) formation efficiency and intracellular multiplication. We first identified 13 (1.2%) relapse, 7 (0.7%) persistent, and 3 (0.3%) reinfection patients from 1,046 survivors. Each of the 20 within-host strain pairs from patients with relapse and persistent infections shared the same genotype, suggesting that the subsequent isolates arise from the infecting isolate. Logistic regression analysis of clinical data revealed regimen and duration of oral antibiotic therapies as risk factors associated with relapse and persistent infections. WGS analysis demonstrated 17 within-host genetic alteration events in 6 of 20 paired isolates, including a relatively large deletion and 16 single-nucleotide polymorphism (stocktickerSNP) mutations distributed across 12 genes. In 1 of 20 paired isolates, we observed significantly increased cell-to-cell fusion and intracellular replication in the second isolate compared with the initial isolate from a patient with persistent infection. WGS analysis suggested that a non-synonymous mutation in the *tssB-5* gene, which encoded an essential component of the type VI secretion system, may be associated with the increased intracellular replication and MNGC formation efficiency of the second isolate of the patient. This information provides insights into genetic and phenotypic alterations in *B. pseudomallei* in human melioidosis, which may represent a bacterial strategy for persistent and relapse infections.

## Introduction

Melioidosis is a fatal tropical infectious disease caused by a Gram-negative bacillus *Burkholderia pseudomallei*, a motile saprophyte surviving in various environmental niches such as soil, water, and plant rhizosphere (Seng et al., 2019). Melioidosis is most prevalent in Southeast Asia and Northern Australia; yet, it is estimated that 165,000 human cases and 89,000 deaths per year occur worldwide (Limmathurotsakul et al., 2016). The mortality rates in Southeast Asia and Northern Australia are approximately 40% (Limmathurotsakul et al., 2010) and 10% (Currie et al., 2010), respectively. Currently, there is no vaccine for melioidosis; however, the disease can be treated with intravenous (IV) ceftazidime or meropenem, followed by oral trimethoprim/sulfamethoxazole for 3–6 months. However, delayed treatment due to low awareness and late diagnosis can often lead to poor outcomes (Hinjoy et al., 2018; Wiersinga et al., 2018).

Treatment of melioidosis is difficult because *B. pseudomallei* possesses various virulence factors to invade host cells, providing a sheltered survival niche to protect from the circulating immune system and antibiotics, which may result in severe disease and relapse infection (Finlay and McFadden, 2006; Limmathurotsakul et al., 2006; Wiersinga et al., 2018; Lennings et al., 2019). Relapse is a subgroup of recurrent infection that is defined as when a patient had signs of infection and remained culture positive at subsequent episodes for the same *B. pseudomallei* clone as initial episode (Chaowagul et al., 1993; Limmathurotsakul et al., 2006; Sarovich et al., 2014; Rachlin et al., 2016; Halim et al., 2017; San Martin et al., 2018). During 1986–1991, 15% of melioidosis cases were identified as relapses at Sunpasitthiprasong Hospital, Ubon Ratchathani, Northeast Thailand (Chaowagul et al., 1993). At the same hospital, when the study period was extended from 1986 to 2005, the relapse rate was reduced to 9.7% (Limmathurotsakul et al., 2006). However, the current situation of relapse cases in the whole of Northeast Thailand is unknown. In addition to relapse, some melioidosis patients have no evidence of clinical response to oral therapy and remained culture positive for the same *B. pseudomallei* clone during antibiotic treatment. Although the persistent infection was not well-characterized for melioidosis, we defined these patients as previously described in a study on tuberculosis (TB) (Vargas et al., 2021). Although *B. pseudomallei* can establish and maintain persistent infection in many organs, the number of melioidosis cases with persistent infection has not been reported previously.

During the period between primary and relapse episodes, *B. pseudomallei* may undergo dramatic genomic and transcriptomic changes to exist in hostile niche and contribute to persistent or relapse episodes (Hayden et al., 2012; Viberg et al., 2017; Price et al., 2018; Pearson et al., 2020). Reactivation of latent infection may be triggered by various stimuli including bacterial factors and host immunosuppression, leading to subsequent relapse. Thus, it is necessary to find within-host phenotypic and genetic alterations in relapse and persistent patients in order to understand the strategy for survival and pathogenesis of this bacterium. *B. pseudomallei* can intracellularly replicate and spread to neighboring cells *via* cell-to-cell fusion, resulting in the formation of multinuclear giant cells (MNGCs) that ultimately overwhelm the host cells (Toesca et al., 2014; Lennings et al., 2019). Although previous studies have focused on the within-host alterations to increase antibiotic resistance, the changes in intracellular replication and cell-to-cell fusion capabilities of *B. pseudomallei* are not well-investigated. Previous studies have reported the reduction of ceftazidime, doxycycline, trimethoprim/sulfamethoxazole, chloramphenicol, ofloxacin, and amoxicillin-clavulanic susceptibilities in a few relapse *B. pseudomallei* isolates when compared with the initial isolates (Hayden et al., 2012; Viberg et al., 2017; Fen et al., 2021). These studies demonstrated that the reduction of antibiotic susceptibility was associated with within-host mutations in multiple genes, such as in beta-lactamase class A (*penA*), BpeEF-OprC operon, and TetR family (*amrR*). In addition, a previous study on whole-genome-sequencing (WGS) of 69 *B. pseudomallei* colonies collected from specimens of seven body sites of an acute melioidosis patient identified 14 within-host mutation events (8 SNPs and 6 small indels) in several genes (Limmathurotsakul et al., 2014) but did not characterize the phenotypic changes of bacteria in patients.

Coupled with bacterial genetic alternations during relapse and persistent infections, a better understanding of risk factors for relapse and persistent infections is required to improve patient outcomes. A previous study of 89 patients in a single hospital since 2004 has identified types of antibiotics and duration of oral antimicrobial therapy as the most important determinants of relapse, followed by positive blood culture results and multifocal distribution of the disease (Limmathurotsakul et al., 2006). However, the risk factors associated with relapse, particularly with persistent melioidosis patients in the current situation since 2004, are not well-characterized. Due to the lack of these data, no measures have been developed to eradicate persistent infection of melioidosis.

To investigate the current prevalence and risk factors for relapse and persistent infections of melioidosis in the whole Northeast region of Thailand, we performed a longitudinal prospective study to isolate *B. pseudomallei* from 1,361 melioidosis patients in nine hospitals in this region from July 2015 to December 2018 (Chantratita et al., 2023). We further evaluated MNGC formation, intracellular survival, and WGS of initial and subsequent isolates from the same patients (within-host strains pairs) to analyze within-host phenotypic and genetic changes of this pathogen. The results of this study suggest the potential mechanisms by which persistent infections are maintained in human melioidosis.

## Materials and methods

### Ethical approval

Ethical approval of this study was obtained from the Ethics Committee of the Faculty of Tropical Medicine, Mahidol University (approval number MUTM 2015-002-01, MUTM 2018-039-01, and MUTM 2021-055-01) and of 9 hospitals in Northeast Thailand, including Udon Thani Hospital (approval number 0032.102/318), Khon Kaen Hospital (approval number KE58068), Srinakarin Hospital (approval number HE581259), Nakhon Phanom Hospital (approval number IEC-NKP1-No. 15/2558), Mukdahan Hospital (approval number, MEC 010/59), Roi Et Hospital (approval number 166/2559), Surin Hospital (approval number 9/2559), Sisaket Hospital (approval number SSKH REC No. 034/2560), and Buriram Hospital (approval number BR 0032, 102.3/57).

### Participants and clinical data collection

A prospective observational study was conducted in 9 hospitals in Northeast Thailand from July 2015 to December 2018 (Chantratita et al., 2023). Written consents were obtained from the 1,361 adult male and female patients (age ≥ 15 years) with any specimens taken from any sites positive for *B. pseudomallei* by bacterial culture. Melioidosis patients who were pregnant, receiving palliative care, or incarceration were not enrolled in the study. *B. pseudomallei* were collected on the day of enrolment (day 0), and participants were followed up on days 5, 12, and 28 and every 2 months *via* phone call to collect data on the use of antibiotic therapy, clinical outcomes, and the occurrence of recurrent infections. Demographics and clinical data (age, sex, current illness, symptoms, vital signs, underlying morbidity, diagnosis, laboratory results, antimicrobial therapy, and clinical outcomes) of each participant were collected from medical records and hospital microbiology databases. Clinical and follow-up data were recorded in case report forms (CRFs).

Acute melioidosis was defined as the recent manifestation of melioidosis symptoms with a clinical sample culture growing *B. pseudomallei*, without any evidence of having (i) melioidosis before enrolment and (ii) recurrent infection during follow-up (Currie et al., 2000). Recurrent melioidosis was defined when a patient had (i) signs of infection determined by the attending physician and (ii) remained culture positive for *B. pseudomallei* at subsequent episodes after completion of antibiotic therapy. A patient with recurrent infection with the same *B. pseudomallei* genotype that was collected at the first episode was defined as relapse, whereas a patient who had recrudescence of a different genotype was defined as reinfection or was infected with multiple strains (Chantratita et al., 2023). A patient with no evidence of clinical response to oral antibiotic therapy and who subsequently remained culture positive for *B. pseudomallei* that shared the same genotype clone as the first isolate while undergoing antibiotic therapy was defined as having a persistent infection (Vargas et al., 2021).

### Bacterial collection and identification

Two *B. pseudomallei* isolates were longitudinally collected from any positive specimens of each melioidosis patient at two time points: on the first day of enrolment (first isolate) and on the day of the recurrent or persistent episode (second isolate). The identification of *B. pseudomallei* was performed in the microbiology laboratory at each hospital using culture, Gram staining, immunofluorescence assay (IFA), latex agglutination, and standard biochemical tests (Duval et al., 2014; Dulsuk et al., 2016). The bacterial identification was confirmed at the Faculty of Tropical Medicine, Mahidol University, using Matrix-Laser Absorption Ionization Mass Spectrometry (MALDI-TOF MS) as previously described (Suttisunhakul et al., 2017). Bacteria were sub-cultured on Ashdown agar plates, incubated at 37°C overnight, and stored at −80°C in trypticase soy broth containing 20% glycerol.

### Pulse-field gel electrophoresis (PFGE)

Genotypes of *B. pseudomallei* paired isolates collected at the first and second episodes of melioidosis were characterized by PFGE as previously described (Chantratita et al., 2008).

### Multinucleated giant cell formation assay

To investigate the phenotypic changes of *B. pseudomallei*, we compared the ability to induce cell-to-cell fusion between the first and second isolates from each relapse or persistent patients using a modified MNGC assay (Sangsri et al., 2020). Briefly, A549 cells were seeded at 1.5 × 10^4^ cells per well into a 96-well plate and incubated at 37°C in 5% CO_2_ overnight. The cells were washed with PBS and infected with *B. pseudomallei* at MOI of 30:1 and incubated at 37°C for 2 h. The infected cells were washed with PBS and re-incubated with fresh RPMI medium (Gibco BRL, Grand Island, NY, USA) supplemented with 10% heat-inactivated fetal bovine serum (FBS) (HyClone, USA) and 250 μg/ml of kanamycin (Gibco BRL). At 10 h post-infection, the cells were washed and fixed with 4% paraformaldehyde for 30 min and stained with Giemsa stain (Merck KGaA, Darmstadt, Germany). The cells were then examined under a light microscope. The number of MNGC cells was analyzed using the ImageJ software version 1.52n.^1^ An MNGC was defined as a cell having three or more nuclei (Whiteley et al., 2017). For each field, the total number of nuclei in MNGCs and the total number of MNGCs were counted. The MNGC efficiency was calculated using the formula: (number of nuclei in MNGCs/total number of nuclei) × 100. The MNGC size was calculated as follows: the total number of nuclei in MNGCs/total number of MNGCs. The MNGC formation assay was performed in three independent experiments, each was performed in triplicate.

### Intracellular survival assay

An intracellular bacterial survival assay was performed to compare the ability of *B. pseudomallei* pairs to grow within the host cells. The assay was performed in two independent experiments as previously described (Saiprom et al., 2020), but with some modifications. Briefly, A549 cells were seeded at 1.5 × 10^4^ cells per well into a 96-well plate, washed with PBS, and infected with *B. pseudomallei* at MOI of 30:1 as described in the MNGC assay. The infected cells were washed with PBS and re-incubated with fresh RPMI medium supplemented with FBS (HyClone) and 250 μg/ml of kanamycin (Gibco BRL). The cells were then washed and lysed with 0.1% v/v Triton X-100 (Sigma) and performed colony count at 2, 4, 6, 8, and 10 h post-infection. Each experiment was performed in triplicate.

### Immunostaining and confocal microscopy

The intracellular growth of *B. pseudomallei* and cell-to-cell fusion were validated by immunostaining and visualization using confocal microscopy (Sangsri et al., 2020). Briefly, A549 cells were seeded at 1 × 10^6^ cells per well on a sterile glass coverslip in a 6-well plate and incubated at 37°C in 5% CO_2_ overnight. The cells were washed with PBS and infected with *B. pseudomallei* at MOI of 30:1 and incubated for 2 h. The infected cells were then washed with PBS and re-incubated with fresh RPMI medium supplemented with 10% FBS and 250 μg/ml of kanamycin. At 8 h post-infection, the cells were washed and fixed with 4% paraformaldehyde for 30 min and permeabilized with 0.5% Triton X-100 for 30 min. The permeabilized cells were incubated with 2.5 μg/ml of 4B11 monoclonal antibody specific to *B. pseudomallei* capsular polysaccharide at 37°C for 1 h. The cells were further incubated with goat anti-mouse IgG conjugated with Alexa Fluor 488, phalloidin conjugated with Alexa Fluor 647, and Hoechst 33258, at dilution of 1:1,000, to stain *B*. *pseudomallei*, cytoplasm, and nucleus, respectively. The stained cells were then washed three times with PBS. The coverslips were mounted on glass slides using 8 μl of ProLong Gold antifade reagent (Invitrogen). Staining cells and bacteria were imaged with a laser scanning confocal microscope (LSM 700; Carl Zeiss, Germany) using a 20 × and 100 × objective lens with oil immersion and Zen software (2010 edition, Carl Zeiss). The excitation and emission wavelengths for Alexa Fluor 488, Hoechst 33258, and Alexa Fluor 647 were 496/519, 352/461, and 594/633, respectively. The number of intracellular survival bacteria was calculated from 20 images (by 20 × objective lens) of two independent experiments using the ImageJ software.

### Bacterial growth curve analysis

Growth curve analysis was performed to determine the ability of *B. pseudomallei* to grow in an enrichment medium as previously described (Saiprom et al., 2020). A single colony of *B. pseudomallei* was inoculated in 3 ml of Luria-Bertani (LB) broth and incubated at 37°C with shaking at 200 rpm for 18 h. Culture bacteria were centrifuged at 12,000 rpm for 5 min, washed with PBS, and resuspended with PBS. The bacterial suspension adjusted to the optical density (OD) at 600 nm to obtain a bacterial concentration of approximately 1 × 10^8^ CFU/ml. The bacterial suspension was added to 10 ml of LB broth to make a final concentration of 1 × 10^5^ CFU/ml. Cultures were incubated at 37°C with shaking at 200 rpm. The viable count was performed at 0, 2, 4, 6, 8, 10, 12, and 24 h post-infection. The growth curve was determined from two independent experiments.

### DNA extraction and whole-genome sequencing

Bacterial genomic DNA was extracted from *B. pseudomallei* isolates using QIAamp DNA Mini Kit (Qiagen, Germany). Genomic DNA was subjected to the 150-base-read library preparation. WGS was performed in the Illumina HiSeq2000 system with 100-cycle paired-end runs at Wellcome Sanger Institute, Cambridge, UK. To enhance the sensitivity and specificity of within-host mutation analysis, the genomic DNA of a paired isolate from a patient P17 (whose second isolate showed a dramatic change in MNGC formation compared with the first isolate) was analyzed using a long-read sequencing on a MinION sequencer (Oxford Nanopore Technologies, Oxford, UK). FastQC version 0.11.3^2^ was used to check the quality of all genomes. The accession number for all genomes is listed in Supplementary data 1.

### Genome assembly and annotation

The *de novo* assembly of short-read data was performed for all *B. pseudomallei* genomes using Velvet version 1.2.10 (Zerbino and Birney, 2008). For each genome, this resolved into an average of 132 contigs (range 74–210 contigs), thereby providing a high resolution for detecting genomic alternations between the primary and relapse and persistent strains. The complete hybrid assembly was performed on long- and short-read sequence data of a paired isolate from P17 using Unicycler version 0.8.4 (Wick et al., 2017). The complete hybrid assembly was used as an internal reference for enhancing the sensitivity and specificity of within-host mutation analysis of isolates from P17. Our hybrid assembly produces two contigs, corresponding to two chromosomes of *B. pseudomallei*. All assemble genomes were annotated by employing Prokka version 1.13.4 (Seemann, 2014) using *B. pseudomallei* K96243 genome as a reference. This led to an average of 5,925 genes being annotated per genome (range 5,809–6,089), falling within the same range of previous literature (Holden et al., 2004; Nandi et al., 2010; Chewapreecha et al., 2019).

### Within-host mutation analysis

We studied within-host alterations of all paired isolates collected from relapse and persistent patients by comparing genomes of *B. pseudomallei* pairs isolated from the same patients. Variants including SNPs and indels were called from the genomic comparison using Snippy version 4.6.0.^3^ To avoid false variants, we filtered out reads with < 10 read depth and found a frequency of < 90%. All variants identified by Snippy were curated manually by visualizing with the Tablet software.^4^ The functions of mutated genes were annotated using Clusters of Orthologous Group (COG) terms of *B. pseudomallei* K96243 available on the *Burkholderia* Genome Database (Winsor et al., 2008).

### Statistical analysis

Data were analyzed using the R version 4.0.4 and GraphPad Prism 10 software (GraphPad Inc.). Firth logistic regression (Puhr et al., 2017) was used to analyze risk factors for relapse and persistent infections using R-package logistf^5^. The Mann–Whitney *U* test was performed to analyze the differences in the median of MNGC efficiency, MNGC size, and the number of intracellular bacteria between the first and second isolates from patients with relapsed or persistent infections.

## Results

### Patient characteristics and risk factors for relapse and persistent melioidosis

**FIGURE 1:**
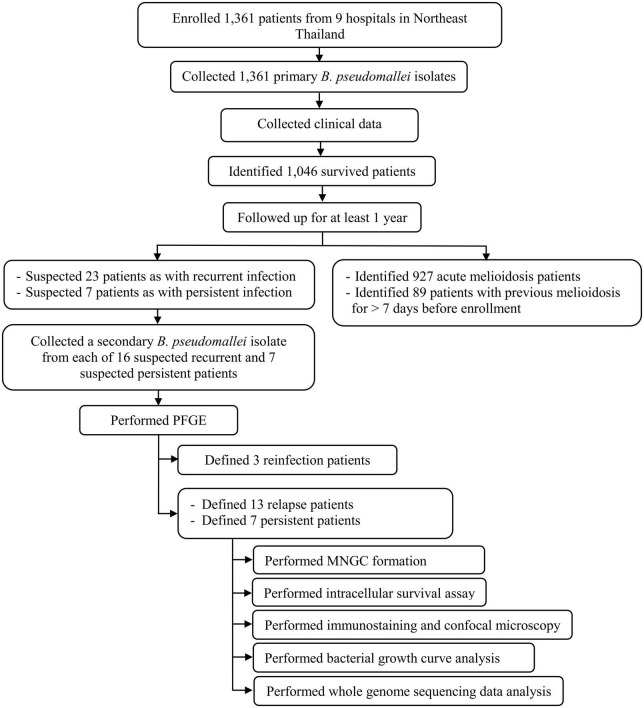
Flow diagram of study procedures, number of patients, and bacterial isolates used in our cohort.

We enrolled 1,361 melioidosis patients from 9 hospitals in Northeast Thailand, of whom 1,046 (76.8%) survived and were discharged from the hospital, allowing us to follow up to investigate the prevalence of relapse, persistence, and reinfection. Of all 1,046 patients, 927 patients had acute melioidosis, 89 patients were suspected to have a history of melioidosis at least 8 days before enrolment, while 23 and 7 patients were suspected to have recurrent and persistent infections, respectively. Of the 23 suspected recurrent patients, we excluded 7 patients, since the subsequent *B. pseudomallei* isolates were not collected from the recurrent episode. Therefore, we analyzed the remaining 16 suspected recurrent patients and 7 suspected persistent patients using PFGE, MLST, and WGS (Figure 1). Genotyping analyses of all *B. pseudomallei* paired isolates from these patients revealed 13 (1.2%) patients with relapse, 7 (0.7%) with persistent infection, and 3 (0.3%) with reinfection or polyclonal infection (Figure 1; Supplementary data 1). The genomic evidence supporting the relapse and persistent patients is given in Supplementary data 2. The median duration of relapse and persistent was 245 days (range 164–518, IQR = 181–278) and 74 days (range 38–174, IQR = 68–103), respectively.

Relapse and persistent infections represent treatment failure in melioidosis. Understanding the risk factors associated with relapse and persistent infections is fundamental to the control of infection. Since the number of relapses and persistent infections was low, we performed Firth logistic regression to identify risk factors for the combined number of patients with relapse and persistent infections (*N* = 20). Firth logistic regression is an approach for the analysis of rare event data. Results in Table 1 demonstrated that the regimen and duration of oral antibiotic therapies were the factors associated with relapse and persistent infections (*P* = 0.005 and *P* = 0.031, respectively). For a regimen of oral antibiotic therapy, patients who were treated with amoxicillin, ciprofloxacin, clindamycin, and/or rifampicin were more presented in relapse and persistent than patients with acute infection [7/20 (35.0%) vs. 106/927 (11.4%)]. Similarly, patients who were treated with trimethoprim/sulfamethoxazole were more presented in acute infection than patients with relapse and persistent infections [693/927 (74.8%) vs. 10/20 (50.0%)]. For the duration of antibiotic therapy, patients who received any of these oral antibiotics (trimethoprim/sulfamethoxazole, doxycycline, augmentin, amoxicillin, ciprofloxacin, clindamycin, and rifampicin) for 3–6 months were not likely to be at risk of relapse and persistent than those with acute [8/20 (40.0%) vs. 567/927 (61.2%)]. However, underlying morbidity, clinical manifestation, regimen, and duration of initial IV antibiotic therapy were not associated with the risk of relapse and persistent infections.

**TABLE 1:** Patient characteristics and risk factors for relapse and persistent infections.

Patient characteristics and antibiotic therapy	Acute melioidosis *n* = 927 no. (%)	Relapse and persistent *n* = 20 no. (%)	OR (95% CI)	*P*
**Age (years)**				
15–24	20 (2.2)	2 (10.0)	NA	NA
25–44	172 (18.6)	1 (5.0)	NA	
45–64	545 (58.8)	15 (75.0)	NA	
≥ 65	190 (20.5)	2 (10.0)	NA	
**Sex**				
Male	666 (71.8)	9 (45.0)	NA	NA
Female	261 (28.1)	11 (55.0)	NA	
**Occupation**				
Farmer	594 (64.1)	14 (70.0)	NA	NA
Employee	175 (18.9)	3 (15.0)	NA	
Merchant	10 (1.1)	0 (0.0)	NA	
Others	149 (16.1)	4 (20.0)	NA	
**Underlying morbidity**				
Diabetes mellitus	673 (72.6)	11 (55.0)	2.268 (0.948–5.429)	0.076
Hypertension	271 (29.2)	7 (35.0)	0.532 (0.207–1.369)	
Cancer	22 (2.4)	0 (0.0)	1.110 (0.068–17.888)	
Tuberculosis	54 (5.8)	4 (20.0)	0.252 (0.0869–0.733)	
HIV	12 (1.3)	1 (5.0)	1.194 (0.311–4.584)	
Renal disease	109 (11.8)	2 (10.0)	0.201 (0.035- 1.146)	
**Clinical manifestation**				
Septicemia	714 (77.0)	14 (70.0)	0.737 (0.082–6.615)	0.419
Pneumonia	141 (15.2)	4 (20.0)	0.416 (0.113–1.533)	
Abscess	189 (20.4)	5 (15.0)	0.480 (0.138–1.666)	
Urinary tract infection	48 (5.2)	3 (15.4)	0.186 (0.051–0.674)	
Localized	209 (22.5)	6 (30.0)		
Dissemination	243 (26.2)	5 (25.0)	0.899 (0.102–7.881)	
			1.858 (0.479–7.199)	
**Regimen of initial IV antibiotics**				
Meropenem	308 (33.2)	8 (40.0)	0.622 (0.264–1.464)	0.779
Ceftazidime	857 (92.4)	19 (95.0)	0.858 (0.176–4.163)	
Ceftriaxone	327 (35.3)	7 (35.0)	0.959 (0.400–2.295)	
Augmentin	34 (3.6)	2 (10.0)	0.299 (0.080–1.117)	
Azithromycin	51 (5.5)	0 (0.0)	2.467 (0.179–34.001)	
Clindamycin	238 (25.7)	5 (25.0)	1.070 (0.418–2.740)	
Bactrim	87 (9.4)	1 (5.0)	1.394 (0.286–6.792)	
Other antibiotics	382 (41.2)	6 (30.0)	1.555 (0.644–3.753)	
**Duration of initial IV antibiotic therapy**				
< 10 days	260 (28.0)	5 (25.0)	0.964 (0.579–1.605)	0.889
10–14 days	157 (16.9)	4 (20.0)		
> 14 days	451 (48.6)	10 (50.0)		
**Regimen of oral antibiotic therapy**				
TMP-SMX	693 (74.8)	10 (50.0)	2.090 (0.821–5.319)	0.005**
Doxycycline	51 (5.5)	2 (10.0)	0.325 (0.083–1.266)	
Augmentin	34 (3.7)	3 (15.0)	0.332 (0.084–1.307)	
Other antibiotics^a^	106 (11.4)	7 (35.0)	0.273 (0.103–0.722)	
**Duration of oral antibiotic therapy**				
< 3 months	360 (38.8)	7 (35.0)	2.935 (1.143–7.535)	0.031*
3–6 months	567 (61.2)	8 (40.0)		
> 6 months	36 (3.9)	1 (5.0)		

*Significant levels by Firth logistic regression at *P* < 0.05.

**Significant levels by Firth logistic regression at *P* < 0.01.

^a^Amoxicillin, ciprofloxacin, clindamycin, and/or rifampicin.

### Identification of the persistent *B. pseudomallei* isolate DREX512 with increased cell-to-cell fusion ability

**FIGURE 2:**
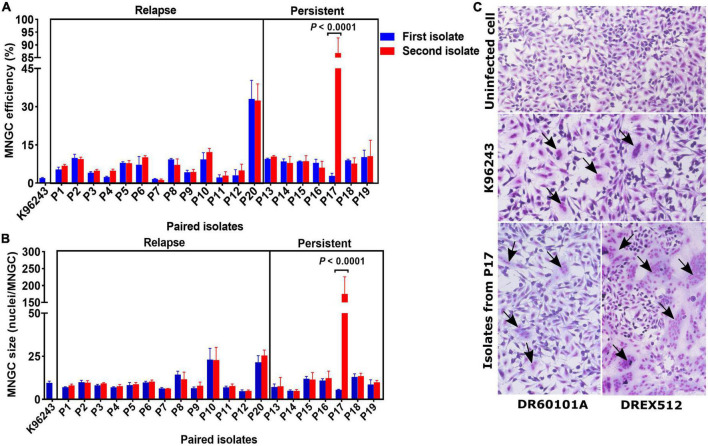
Multinucleated giant cell formation (MNGC) formation in A549 cells infected with paired *Burkholderia pseudomallei* isolates from 13 relapse and seven persistent patients. **(A)** Comparison of MNGC efficiency between the first and second isolates. **(B)** Comparison of MNGC size between the first and second isolates. **(C)** Giemsa stain of uninfected A549 cells, A549 cells infected with reference *B. pseudomallei* K96243, and A549 cells infected with primary DR60101A and persistent DREX512 strains from P17. Black arrows point to the MNGC formation area.

Multinucleated giant cell formation (MNGC) formation assay was performed on paired isolates from all 20 patients to determine the alteration of cell-to-cell fusion ability. We observed variations in MNGC formation ability among *B. pseudomallei* isolates, with overall median MNGC efficiency of 8.0% (range 1.2–88.3%, IQR = 4.6–9.3%) and median size of 9 nuclei/MNGC (range 4.8–177.2, IQR = 6.8–11.7) (Figure 2). Comparison between the paired isolates from all 13 relapse patients showed no significant difference between the first and second isolates on MNGC efficiency and size (Figures 2A, B). All but one pair of the first and second isolates from seven persistent patients showed similar MNGC efficiency and size. Interestingly, the second isolate DREX512 from P17 showed significantly higher MNGC efficiency and size than the first isolate DR60101A (efficiency 88.3 vs. 3.6%, *P* < 0.0001; size 177.2 vs. 5.5, *P* < 0.0001) (Figures 2A–C). DR60101A and DREX512 were isolated 98 days apart. The dramatic change in MNGC efficiency of the second isolate compared with the first isolate suggests that *B. pseudomallei* increased cell-to-cell fusion induction during infection in patient P17.

### Cell-to-cell fusion of DREX512 is associated with intracellular replication

**FIGURE 3:**
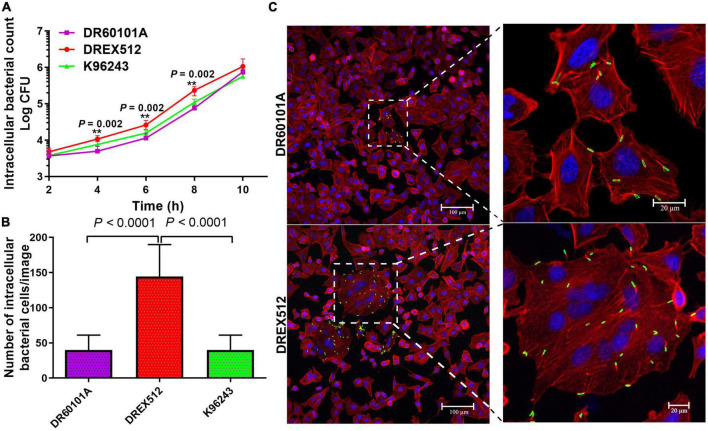
Intracellular survival and immunostaining of A549 cells infected with primary DR60101A and persistent DREX512 strains from P17 and reference *Burkholderia pseudomallei* K96243. **(A)** Intracellular survival at 2, 4, 6, 8, and 10 h post-infection by plate counting. **(B)** Number of bacterial cells per image at 8 h post-infection by confocal microscopy. **(C)** Visualization of infected staining A549 cells by confocal microscopy. Bacteria were stained in green, cytoplasm in red, and DNA in blue.

To determine whether the second isolate from P17 has also changed the ability to grow within the host cells, we performed intracellular survival assays of paired isolates from P17 and reference stain K96243 in A549 cells at 2, 4, 6, 8, and 10 h post-infection. Our experiment demonstrated that the second isolate DREX512 could survive and increased its intracellular growth over time after infection compared with the first isolate DR60101A and *B. pseudomallei* K96243, suggesting that increased cell-to-cell fusion efficiency was related to intracellular replication in DREX512 (Figure 3A). The median number of intracellular bacteria of the second isolate DREX512 was significantly higher than that of the first isolate DR60101A at 4 (10,125 ± 2,125 CFU/ml vs. 5,000 ± 2,350 CFU/ml, *P* = 0.002), 6 (23,875 ± 5,687 CFU/ml vs. 11,250 ± 2,300 CFU/ml, *P* = 0.002), and 8 h (238,750 ± 71,250 CFU/ml vs. 84,500 ± 28,375 CFU/ml, *P* = 0.002) post-infection, but not different at 10 h post-infection (925,000 ± 168,750 CFU/ml vs. 750,000 ± 512,500 CFU/ml, *P* = 0.192). However, we further validated the intracellular growth of bacteria by visualization using confocal microscopy at 8 h post-infection. Our confocal microscopy confirmed that DREX512 formed larger MNGC and multiplied better in the host cells than DR60101A (144 ± 74 vs. 40 ± 23 bacteria cells/image, *P* < 0.0001) and K96243 (median 45 ± 27 bacteria cells/image, *P* < 0.0001) (Figures 3B, C).

### DREX512 did not enhance growth in the enrichment medium

To examine whether the second isolate DREX512 also enhances the growth in the enrichment medium compared with the first isolate DR60101A and the reference strain K96243, we performed two experiments of growth curve analysis of these isolates in LB at 8 time points. Our assay demonstrated that the growth rate of DREX512 was comparable to DR60101A and K96243 at every time point (Supplementary Figure 1). This result indicated that DREX512 normally replicated in LB broth but increased its replication in the host cells.

### WGS analysis revealed multiple within-host mutations of *B. pseudomallei*

**FIGURE 4:**
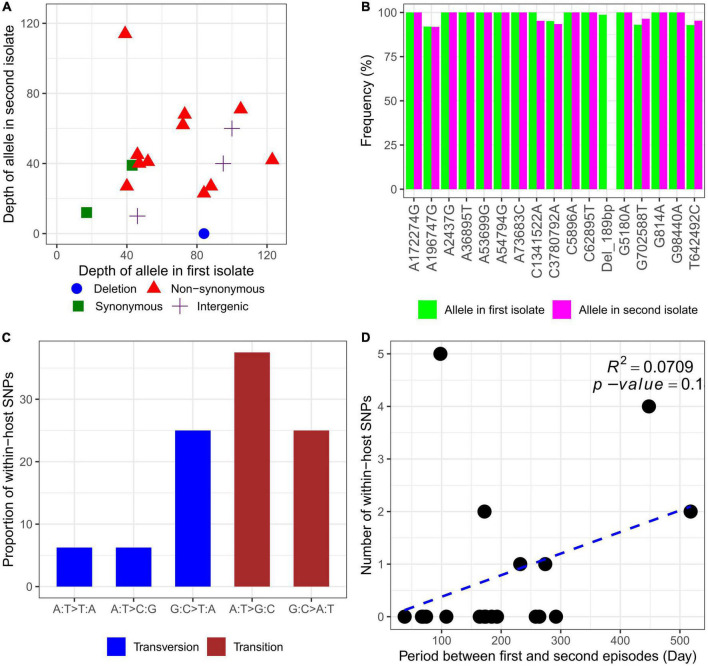
Characteristics of within-host mutations. **(A)** Read depth of alleles in the first and second isolates. **(B)** Frequency of alleles in the first and second isolates. **(C)** Mutational spectrum of within-host SNPs. **(D)** Within-host SNP counts vs. time between isolates collection.

Whole-genome sequencing (WGS) was performed on the genomic DNA extracted from 20 within-host strain pairs obtained from the patients with relapse (13 pairs) and persistent (7 pairs) infections. Using the Snippy and Tablet software, we identified one indel and 16 SNP mutations of 6 of 20 paired isolates collected from 5 of 13 patients with relapse (P1–P3, P10, and P12) and 1 of 7 patients with persistent infection (P17) (Table 2; Figures 4A, B). Therefore, the within-host strain pairs from P4–P9, P11, P13–P16, and P18–P20 shared identical genome. A relatively large indel mutation (189 bp) was detected in the TetR family regulatory protein (*amrR, BPSL1805*) of a paired isolate from a relapse patient (P12). This structural variant was suspected to be associated with decreased susceptibility to meropenem in our previous study (Fen et al., 2021) and is being validated in future studies. Of the 16 SNPs, 11 were non-synonymous, 2 were synonymous, and 3 were in the intergenic region (Figures 4A, B), indicating the occurrence of purifying pressure on synonymous than on non-synonymous variants. The non-synonymous mutations were distributed across 11 genes involved in multiple functions, including transcription (2 genes), general function prediction (3 genes), cell wall biogenesis (1 gene), inorganic ion transport and metabolism (2 genes), energy production and conversion (1 gene), and virulence (1 gene) (Table 2). To further characterized within-host evolution of *B. pseudomallei*, we performed spectrum mutations analysis of the 16 SNPs. We found that A:T > G:C nucleotide transition was the most common (37.5%), while A:T > T:A and A:T > C:G transversion was 6.2% (Figure 4C). Of the SNPs observed in this study, 50% was G:C > A:T transition and G:C > T:A transversion, the patterns of which putatively occur due to oxidative damage to the bacterial DNA (Ford et al., 2011; Dillon et al., 2015).

**TABLE 2:** Characteristics of all within-host mutation in 6 pairs of *Burkholderia pseudomallei* isolates from patients with relapse and persistent infections.

Patient-ID	Infection	Allele in primary isolate	Allele in secondary isolate	Position	Effect	Consequence	Period between the first and second episodes (days)	Product	Function
P1	Relapse	C	A	5896	Non-synonymous	p.Gly54Val	274	DNA-binding protein	Transcription
P2	Relapse	A	G	53699	Synonymous	p.Leu32Leu	172	Transposase	Replication, recombination, and repair
P2	Relapse	G	A	814	Intergenic	NA	172	NA	NA
P3	Relapse	A	G	54794	Intergenic	NA	448	NA	NA
P3	Relapse	A	T	36895	Non-synonymous	p.Asn516Lys	448	Hypothetical protein	NA
P3	Relapse	G	A	98440	Non-synonymous	p.Gly543Glu	448	Hypothetical protein	Function unknown
P3	Relapse	A	G	172274	Non-synonymous	p.Asp115Gly	448	Unknown domain/metalloprotease fusion protein	General function prediction only
P3	Relapse	G	A	5180	Intergenic	NA	448	NA	NA
P10	Relapse	A	G	2437	Synonymous	p.Ser807Ser	232	Non-ribosomal peptide synthetase	Secondary metabolites biosynthesis, transport, and catabolism
P12	Relapse	189 bp		4915.5103	Disruptive inframe deletion		518	TetR family regulatory protein	Transcription
P12	Relapse	A	C	73683	Non-synonymous	p.Thr33Pro	518	Alanyl-tRNA synthetase	General function prediction only
P12	Relapse	C	T	62895	Non-synonymous	p.Gly110Glu	518	UTP-glucose-1-phosphate uridylyltransferase	Cell wall/membrane/envelope biogenesis
P17	Persistent	A	G	196747	Non-synonymous	p.Tyr56His	98	Hydrolase	General function prediction only
P17	Persistent	T	C	642492	Non-synonymous	p.His97Arg	98	Ferric uptake regulator	Inorganic ion transport and metabolism
P17	Persistent	G	T	702588	Non-synonymous	p.Ala53Ser	98	NAD(P) transhydrogenase subunit alpha	Energy production and conversion
P17	Persistent	C	A	1341522	Non-synonymous	p.Asp61Tyr	98	Type VI secretion system protein (TssB)	Intracellular survival and cell-to-cell fusion
P17	Persistent	C	A	3780792	Non-synonymous	p.Ala133Glu	98	Chromate transporter	Inorganic ion transport and metabolism

For all 20 patients, the mutation rate ranged from 0 to 18.6 SNPs per genome per year (Supplementary data 3). However, an average mutations rate for all within-host strain pairs was not estimated, since an increase in within-host SNPs was not related to the time between isolates collection (*R*^2^ = 0.0709, *P* = 0.1) (Figure 4D). We observed that paired isolates from P17 had the highest number of SNPs (5 SNPs) with a short period of time between isolates collection (98 days). The patient was previously diagnosed and completely treated for TB. For the first episode, the patient was admitted with acute respiratory failure and finally diagnosed with pneumonia melioidosis. The patient received IV ceftazidime for 10 days and was discharged to home 10 days after admission. The patient was prescribed ciprofloxacin for 180 days as oral antibiotic therapy. At 60 days after discharge, the patient was reported not responding to therapy and was readmitted to the same study hospital with pneumonia melioidosis at 98 days after the first admission. Although the IV antibiotic therapy data were not collected during the second episode, the patient was reported to receive oral ciprofloxacin for 180 days which was provided during the first episode. The first and second *B. pseudomallei* isolates were collected from sputum at the first and second episodes, respectively. After the second episode of admission, the patient did not respond to therapy and was readmitted two times to the community hospital. Finally, the patient died 332 days after the first admission or 231 days after readmission at the persistent episode.

Whole-genome sequencing (WGS) analysis of the paired isolates from P17, which differed in intracellular replication and cell-to-cell fusion, identified 5 non-synonymous mutations in 5 different genes, including chromate transporter (*chr, BPSL0285*), hydrolase (*BPSL3337*), NAD (P) transhydrogenase subunit alpha (*BPSL2887*), ferric uptake regulator (*fur*, *BPSL2943*), and type VI secretion system (T6SS-5) protein TssB-5 (*tssB-5*, *BPSS1496*) (Table 2). Since the indel and structural mutations were not detected, we hypothesized that the 5 non-synonymous mutations may play a role in the phenotypic alternations in this strain. The functions of chromate transporter, hydrolase, NAD (P) transhydrogenase subunit alpha, and ferric uptake regulator proteins were not obviously related to within-host infectivity or pathogenesis of *Burkholderia* species (Table 2). The enhancement of intracellular replication and cell-to-cell fusion in a persistent isolate of P17 may not be related to the non-synonymous mutations of these 4 genes. Interestingly, we found a non-synonymous mutation in *tssB-5.* This gene encodes an essential component of T6SS-5, a virulent factor of *B. pseudomallei*, involved in intracellular replication and MNGC formation (Pilatz et al., 2006; Burtnick et al., 2011; Chen et al., 2011; French et al., 2011; Hopf et al., 2014; Schwarz et al., 2014; Toesca et al., 2014; Nguyen et al., 2018). We postulated that a non-synonymous mutation in the *tssB-5* gene shifted *B. pseudomallei* to have stronger intracellular survival and MNGC phenotypes. Unfortunately, according to biosafety regulation, *tssB-5* is a highly restricted pathogenic sequence of *B. pseudomallei* K96243 and *Burkholderia mallei* ATCC 23344. We could not synthesize the *tssB-5* mutant fragment for mutagenesis to investigate the effect of this mutation on intracellular replication and cell-to-cell fusion of the second isolate DREX512 from P17.

## Discussion

*Burkholderia pseudomallei* possesses various virulence factors to overcome immune response and antibiotic therapy, facilitating relapse and persistent infections (Finlay and McFadden, 2006; Limmathurotsakul et al., 2006; Wiersinga et al., 2018; Pearson et al., 2020). In this study, we investigated the prevalence of relapse and persistent melioidosis as well as the associated risk factors, in Northeast Thailand. We also studied the phenotypic and genetic alterations of *B. pseudomallei* during persistent in melioidosis patients. We found a low rate of relapse and persistent infections was associated with the efficiency of oral trimethoprim/sulfamethoxazole therapy. We observed the phenotypic and genetic alterations that may involve in the enhancement of the within-host persistent ability of *B. pseudomallei*.

Our multicenter study during 2015–2018 defined 1.2% of patients as relapsing and 0.7% as having persistent infection. Relapse melioidosis was previously reported in endemic regions, including 4.3% in Darwin Australia (Sarovich et al., 2014), 3.7% in South India (Halim et al., 2017), 4.9% in the Philippines (San Martin et al., 2018), and 0.7% in Laos (Rachlin et al., 2016). In Northeast Thailand, 15% of cases between 1986 and 1991 were identified as relapses, but the rate reduced to 9.7% when the study was prolonged from 1986 to 2005 (Chaowagul et al., 1993; Limmathurotsakul et al., 2006). Our results provide evidence for a low relapse rate compared with the previous report during 1986–2005 in Northeast Thailand, which may indicate the improvement of treatment in this hyperendemic region.

Currently, melioidosis is initially treated with IV ceftazidime or meropenem for 10–14 days. After initial IV therapy, the patient is subsequently treated with trimethoprim/sulfamethoxazole for 3–6 months as the first choice and with augmentin or doxycycline as the second choice (Wiersinga et al., 2018). However, the actual oral antibiotic therapy for melioidosis in Northeast Thailand was not always fixed to trimethoprim/sulfamethoxazole, augmentin, or doxycycline. Patients were found to receive other oral antibiotics such as amoxicillin, ciprofloxacin, clindamycin, or rifampicin either alone or in combination with standard antibiotics for unknown purposes. Although the number of analyzed cases was small, our Firth logistic regression analysis revealed treatment with a non-standard regimen and a short duration of oral antibiotic therapies as the risk factors for relapse and persistent melioidosis, consistent with previous findings (Chaowagul et al., 1993; Currie et al., 2000; Chaowagul et al., 2005; Limmathurotsakul et al., 2006). We observed that most of the non-recurrent melioidosis received trimethoprim/sulfamethoxazole for 3–6 months and that most of the relapse and persistent patients were not prescribed trimethoprim/sulfamethoxazole as oral antibiotic therapy. This result is similar to the report from a previous study in Northeast Thailand revealing a low rate of relapse in patients who received trimethoprim/sulfamethoxazole (Limmathurotsakul et al., 2006). Our study provided evidence to support the efficiency of trimethoprim/sulfamethoxazole for oral antibiotic therapy of melioidosis. Although our analysis suggests that failure to adhere to the treatment guideline could lead to relapse and persistent cases, the results should be treated with caution, given the small sample size of relapse and persistent infection observed in our cohort. Furthermore, we observed that underlying morbidity and initial IV antibiotics were not significantly associated with relapse and persistent infections. This is consistent with previous findings in other Gram-negative bacteria that found no correlation between underlying disease, initial antibiotic therapy, and relapse infection (Surapat et al., 2020). However, there are two limitations of our analysis. First, the duration of initial IV antibiotic therapy may be not true since some patients were referred from community hospital and then referred back to their original hospitals for ongoing treatment, potentially including ongoing IV therapy. Second, the duration of follow up time varied and some patients were lost to follow up. Some of them may develop a recurrent or persistent infection during the lost follow up time, then they may be misclassified as acute melioidosis.

Investigation of within-host phenotypic alteration is crucial to understand the pathogenesis and virulence of *B. pseudomallei*. Our study demonstrated dramatic within-host adaptive changes in intracellular replication and cell-to-cell fusion capabilities of the second isolate from a persistent patient (P17) compared with the first strain. Although the function of cell-to-cell fusion in the pathogenesis of melioidosis is not yet well-determined, *B. pseudomallei* is capable of inducing MNGC in lung tissue of melioidosis patients and infected mice (Wong et al., 1995; Conejero et al., 2011). In addition, deletion of *T6SS-5* genes drastically decreased intracellular replication, cell-to-cell fusion, and virulence of *B. pseudomallei* in mammalian models of acute infection (Pilatz et al., 2006; Burtnick et al., 2011; French et al., 2011; Hopf et al., 2014; Schwarz et al., 2014; Toesca et al., 2014; Saiprom et al., 2020). Thus, it is possible that the dramatic within-host adaptive changes in intracellular replication and cell-to-cell fusion may enhance the virulence *in vivo*, which could influence the outcome of P17. This patient was reported to not responding to antibiotic therapy and died 231 days after persistent episodes. Our finding is in concordance with the previous study in *Pseudomonas aeruginosa* that reported the association between a high rate of intracellular survival and the increase in *in vivo* persistence in a mouse model of urinary tract infection (Penaranda et al., 2021). Nevertheless, the finding of increased MNGC efficiency of the second isolate in our case is different from a report of the virulent attenuation in subsequent clinical isolates compared with early isolates of a chronic relapse patient in Australia (Pearson et al., 2020). This inconsistency could be explained by the difference in patient characteristics, antibiotic therapy, and clinical outcomes between our and their studies.

Similar to phenotypic changes, little is known about the within-host mutation of *B. pseudomallei* at short timescales, particularly in a single persistent patient. In our analysis, indel and non-synonymous mutations were found in genes involved in multiple functions, including transcription, general function prediction, cell wall biogenesis, inorganic ion transport, metabolism, energy production and conversion, and virulence. Our study and some previous articles (Hayden et al., 2012; Viberg et al., 2017; Pearson et al., 2020) did not observe a significant within-host mutational density in any genes of *B. pseudomallei*, as detected in *Mycobacterium tuberculosis* (Vargas et al., 2021). However, mutations in the gene homologous to the TetR family transcriptional regulator of *B. pseudomallei* seem to be common. Within-host mutation in this gene was detected by our analysis and also by previous studies (Hayden et al., 2012; Viberg et al., 2017; Pearson et al., 2020), supporting the evidence of positive selection on this gene. Mutations in this gene were previously suspected to be associated with antibiotic resistance (Viberg et al., 2017; Fen et al., 2021).

Our analysis detected a high rate of non-synonymous substitutions, indicating the purifying pressure on synonymous than on non-synonymous variants. The higher rate of within-host non-synonymous mutations was also observed previously in *Burkholderia* species (Lieberman et al., 2011; Hayden et al., 2012; Price et al., 2013; Viberg et al., 2017) and other Gram-negative bacteria such as *P. aeruginosa* (Cottalorda et al., 2021) and *M. tuberculosis* (Vargas et al., 2021). In addition, our spectrum of mutation analysis revealed half of SNP mutations as cytosine deamination (G:C > A:T) and the formation of 8-oxoguanine (G:C > T:A). This pattern has been commonly found in previous studies (Lee et al., 2012; Sung et al., 2012; Zhu et al., 2014; Vargas et al., 2021) and putatively occurred due to oxidative DNA damage (Ford et al., 2011; Dillon et al., 2015).

Previous studies reported the average of within-host mutations rate over time of *Burkholderia* species from 1 to 3.6 SNPs per genome per year (Lieberman et al., 2011; Silva et al., 2016; Pearson et al., 2020). For our study, although the within-host mutation rate ranged from 0 to 18.6 SNPs per genome per year, we did not estimate the average mutation rate for all paired isolates, since the increase in within-host SNPs was not correlated to the time between isolates collection. The disagreement between mutation rate and time between isolates collection may be affected by mutational rate factors such as environmental stress, disease states, and replication (Bjedov et al., 2003; Price et al., 2010; Matic, 2013). Although our study was unable to identify the level of these factors, intra-host environmental stress, disease states, and replication may be varied among our patients.

Regarding the identification of dramatic within-host adaptive changes in paired isolates from P17, the genomic comparison between the first and second isolates revealed 5 non-synonymous mutations in five different genes, including chromate transporter, hydrolase, NAD (P) transhydrogenase subunit alpha, ferric uptake regulator, and type VI secretion system protein TssB-5 (*tssB-5*). Of these genes, the ferric uptake regulator (Fur) protein is a transcription repressor of many genes in response to iron availability to protect bacteria from reactive oxygen species (ROS) damage (Troxell and Hassan, 2013). Fur was found to be required for survival and virulence in animal models for some bacterial pathogens, such as *Actinobacillus pleuropneumoniae*, *Haemophilus influenzae*, *Helicobacter pylori*, *Staphylococcus aureus*, and *Vibrio cholera* (Troxell and Hassan, 2013). This protein was not found to be essential for the infectivity of *Escherichia coli*, *Vibrio vulnificus*, and *P. aeruginosa* (Alice et al., 2008; Porcheron et al., 2014; Pasqua et al., 2017). For *B. pseudomallei*, no clear evidence supports Fur protein as a virulence factor, which could be important for intracellular replication and cell-to-cell fusion, but the supplementation of iron concentration in cell lines was found to be associated with an increase in intracellular survival and MNGC and plaque formation of this pathogen (Amornrit et al., 2012; Butt and Thomas, 2017). Interestingly, a non-synonymous mutation was found in the *tssB-5* gene of T6SS-5. TssB-5 assembles with TssC-5 protein to form a tubular structure that cycles surrounding an inner tube formed by the Hcp (TssD-5) protein sharpened at one end by the TssI-5 (VgrG) and PAAR proteins of T6SS-5 (Nguyen et al., 2018). Several studies characterized T6SS-5 as an inducer of MNGC formation and also a virulence factor of *B. pseudomallei* in mammalian acute infection models (Pilatz et al., 2006; Burtnick et al., 2011; French et al., 2011; Hopf et al., 2014; Schwarz et al., 2014; Toesca et al., 2014). Similarly, a previous mutagenesis study revealed a decrease in intracellular replication and MNGC efficiency in *tssB-5* mutant compared with wild-type *B. pseudomallei* strains (Chen et al., 2011). Thus, T6SS-5 required TssB-5 for exerting its full function on intracellular replication and MNGC formation, and the non-synonymous mutation in this gene may be associated with enhancing intracellular replication and cell-to-cell fusion in paired isolates from P17. Due to the biosafety regulation, we could not synthesize mutant fragments for mutagenesis to describe the in-depth effect of the non-synonymous mutation in the *tssB-5* gene on the cell-to-cell fusion of the second isolate DREX512 from P17. However, it should be noted that the within-host mutations in P17 might be related to individual differences. Data from our study indicate that the different genetic mutations of *B. pseudomallei* pairs occurred in different patients.

## Conclusion

First, we demonstrated the low number of relapses and persistent melioidosis in Northeast Thailand. We then successfully identified the regimen and duration of oral antibiotic therapies as the risk factors for relapse and persistent infections. We observed the dramatic within-host adaptive changes in intracellular replication and cell-to-cell fusion capabilities in a patient with persistent infection. Our WGS analysis revealed multiple within-host mutations including indel and non-synonymous variants. A non-synonymous mutation in the *tssB-5* gene may involve in phenotypic alterations in intracellular replication and cell-to-cell fusion capabilities. Therefore, our study indicates that the human hosts may provide the opportunity for *B. pseudomallei* to mutate and alter their phenotypes that relate to the enhanced virulence. Further studies are required to describe in-depth genotype-phenotype associations in this complex hostile environment.

## Data availability statement

The datasets presented in this study can be found in online repositories. The names of the repository/repositories and accession number(s) can be found in this article/Supplementary material.

## Ethics statement

The studies involving human participants were reviewed and approved by Ethics Committee of the Faculty of Tropical Medicine, Mahidol University and of nine hospitals in Northeast Thailand. Written informed consent to participate in this study was provided by the participants and participants’ legal guardian/next of kin.

## Author contributions

RS, NC, and TW conceived and designed the study. NC, RS, NS, and AD collected and identified bacterial isolates. NC, RP, RS, and AD collected clinical data. NC and RS analyzed the clinical data, performed the experiments, and wrote the manuscript. NC, CC, and JT contributed reagents, materials, and analysis tools. CC, NC, RS, JT, and WC performed whole-genome sequencing. NC, RS, and EB performed bioinformatics analysis. NC was responsible for project administration and supervision. TW, CC, EB, and NC reviewed and edited the manuscript. All authors read and approved the manuscript.

## Footnotes

1
http://rsb.info.nih.gov/ij/

2
https://github.com/s-andrews/FastQC

3
https://github.com/tseemann/snippy

4
https://github.com/cropgeeks/tablet

5
https://github.com/cran/logistf

## Supplementary material

Supplementary Figure 1 Growth curves of primary DR60101A and persistent DREX512 strains from P17 and reference *Burkholderia pseudomallei* K96243 in Luria-Bertani (LB) broth.
