# Supplementary figures and images for: Phenotypic and genetic alterations of Burkholderia pseudomallei in patients during relapse and persistent infections
Source: Front Microbiol. 2023 Feb 6;14:1103297. doi: 10.3389/fmicb.2023.1103297 (PMC9939903; doi:10.3389/fmicb.2023.1103297)

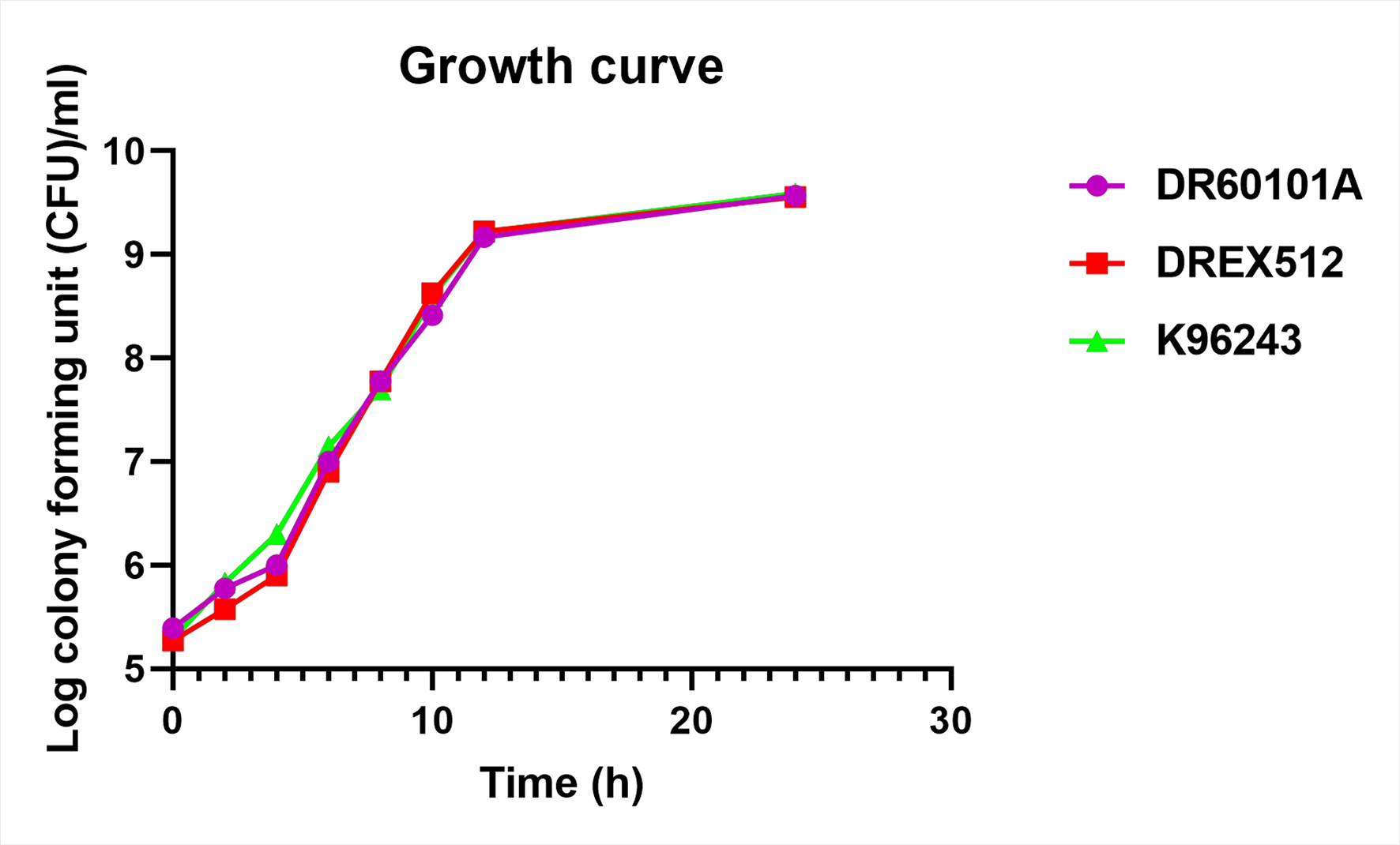

Supplement: Supplementary Figure 1 — Growth curves of primary DR60101A and persistent DREX512 strains from P17 and reference Burkholderia pseudomallei K96243 in Luria-Bertani (LB) broth. [file Image_1.TIFF]
